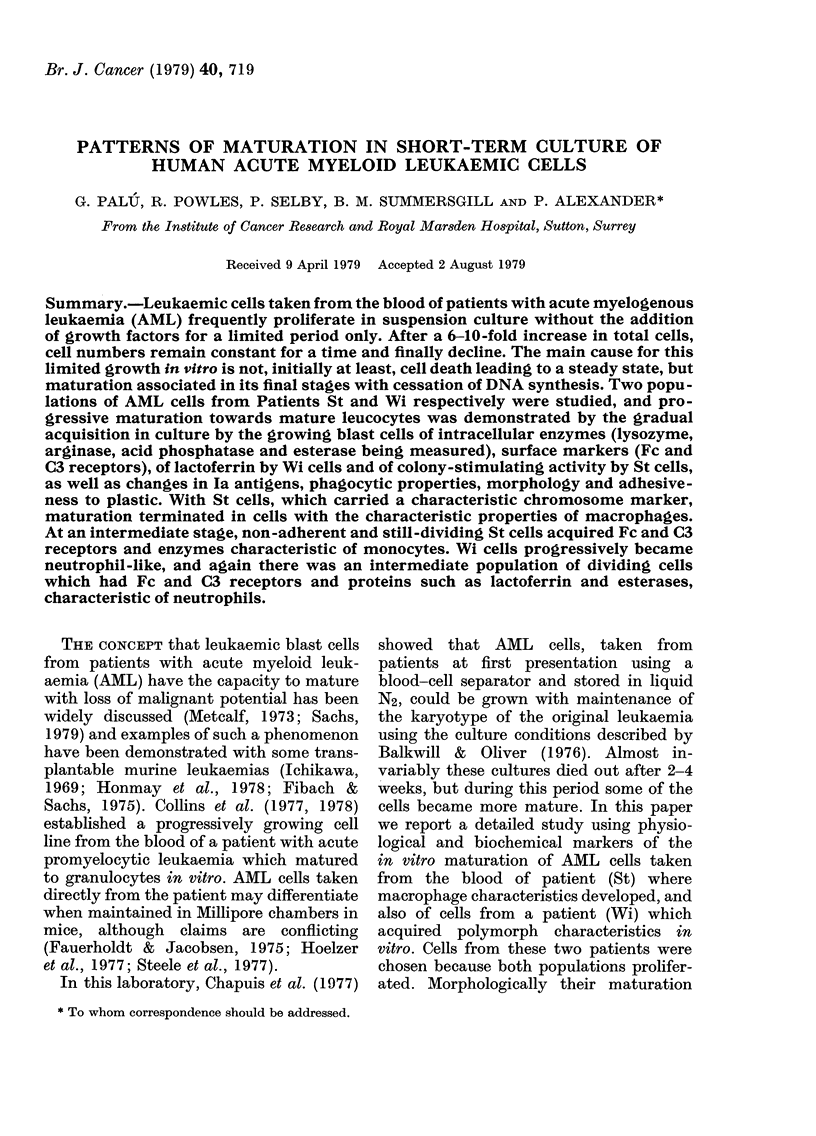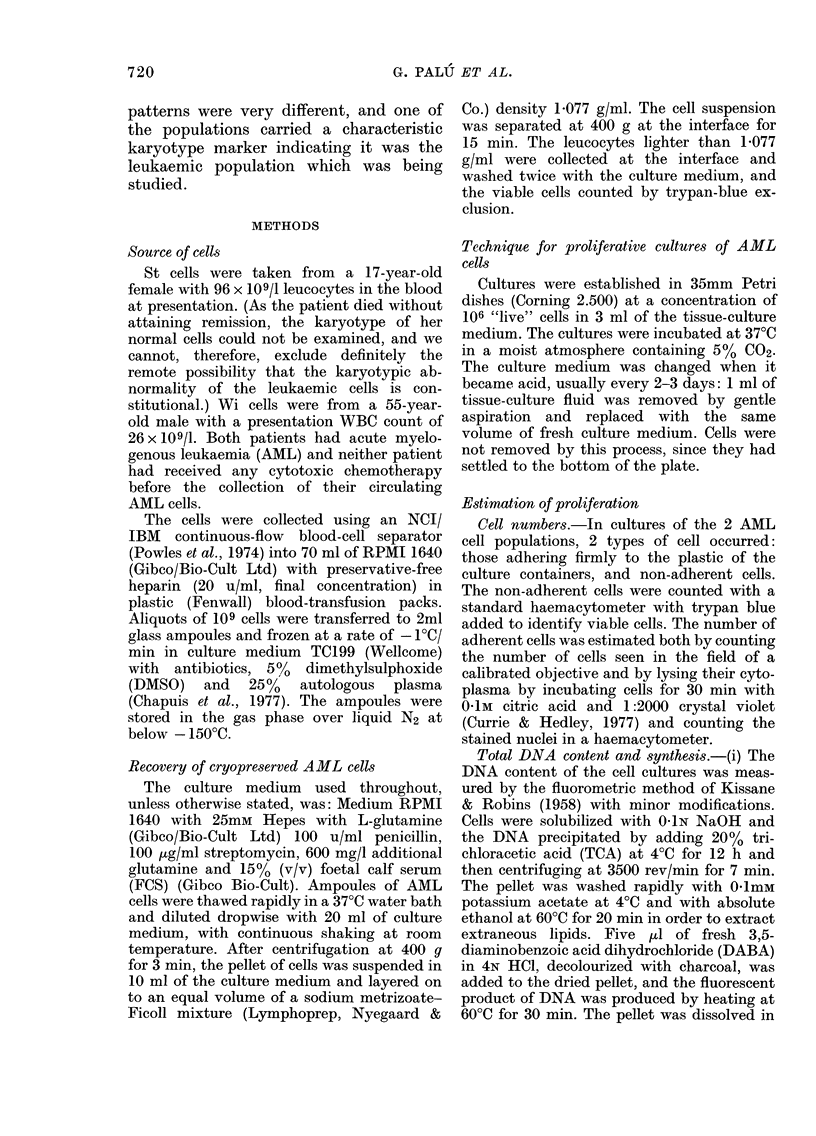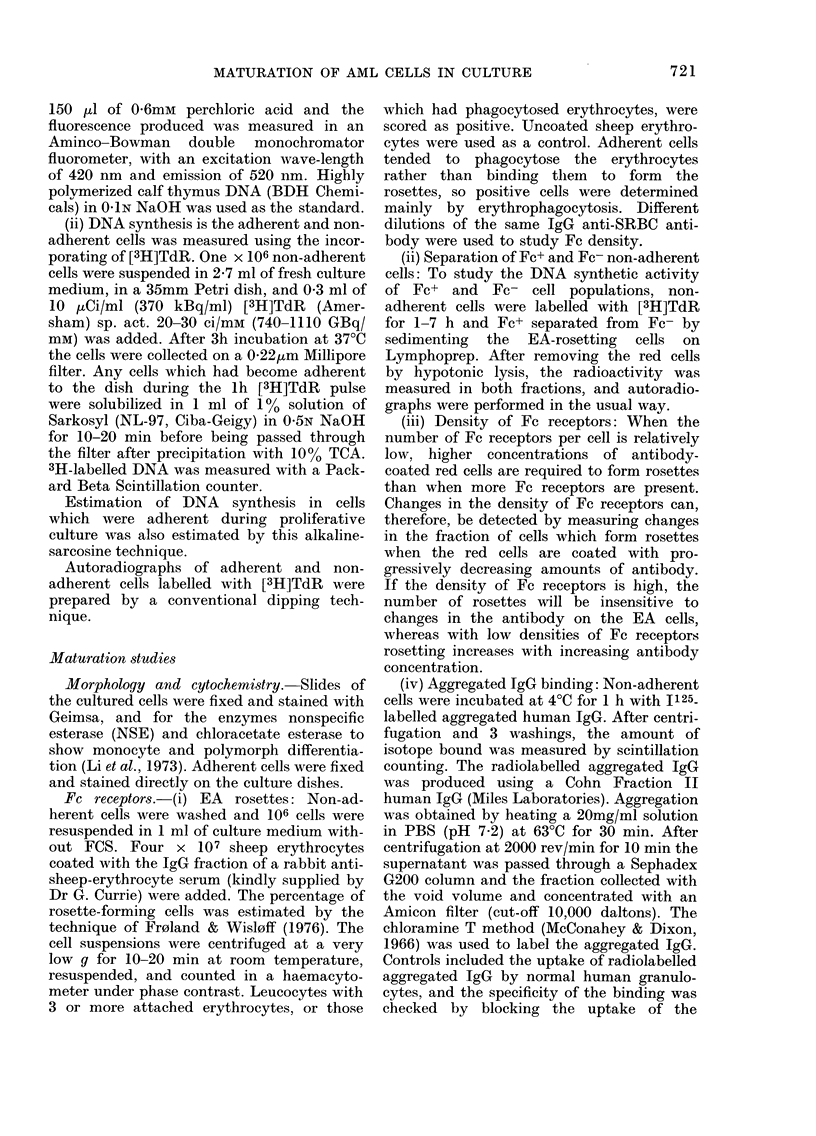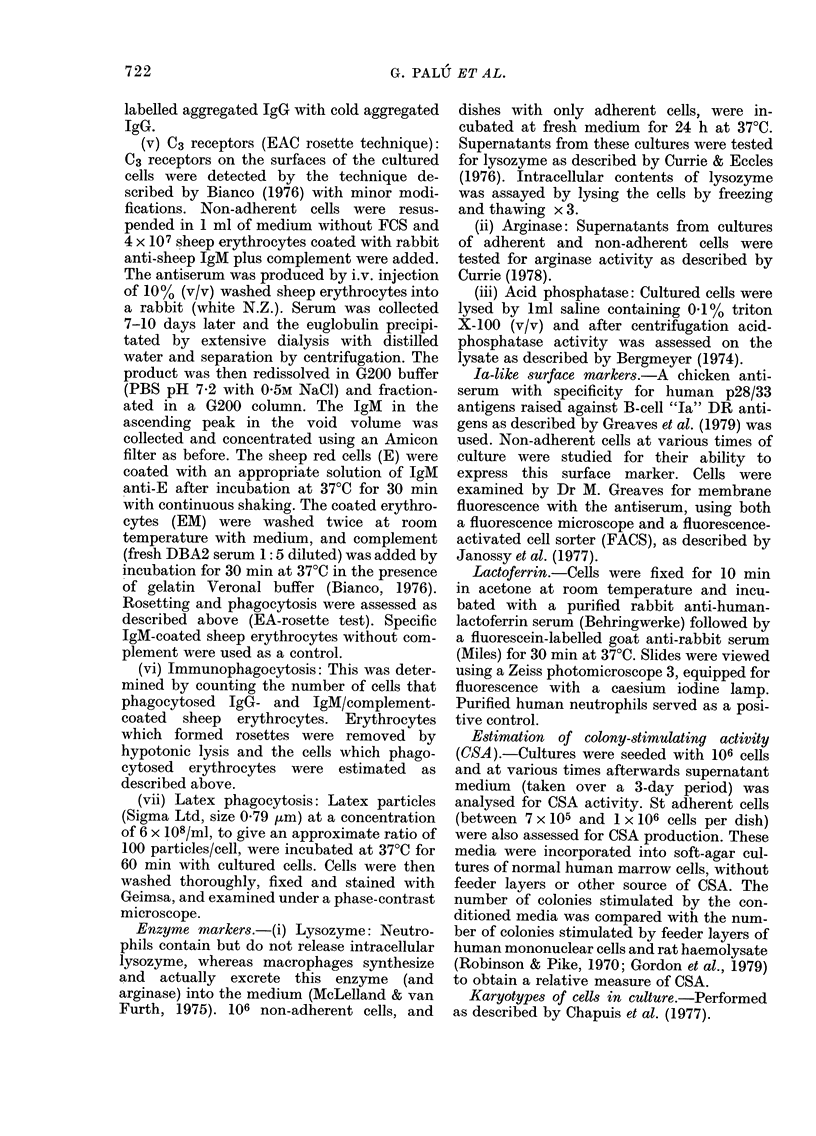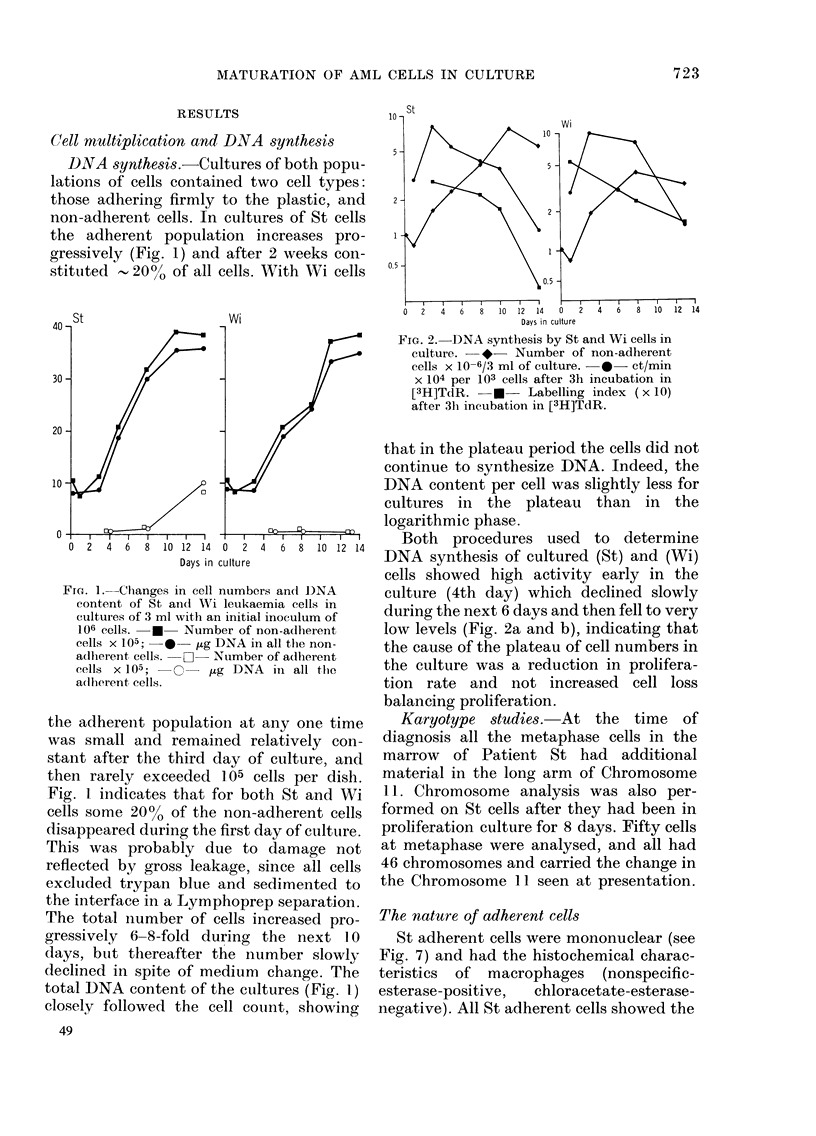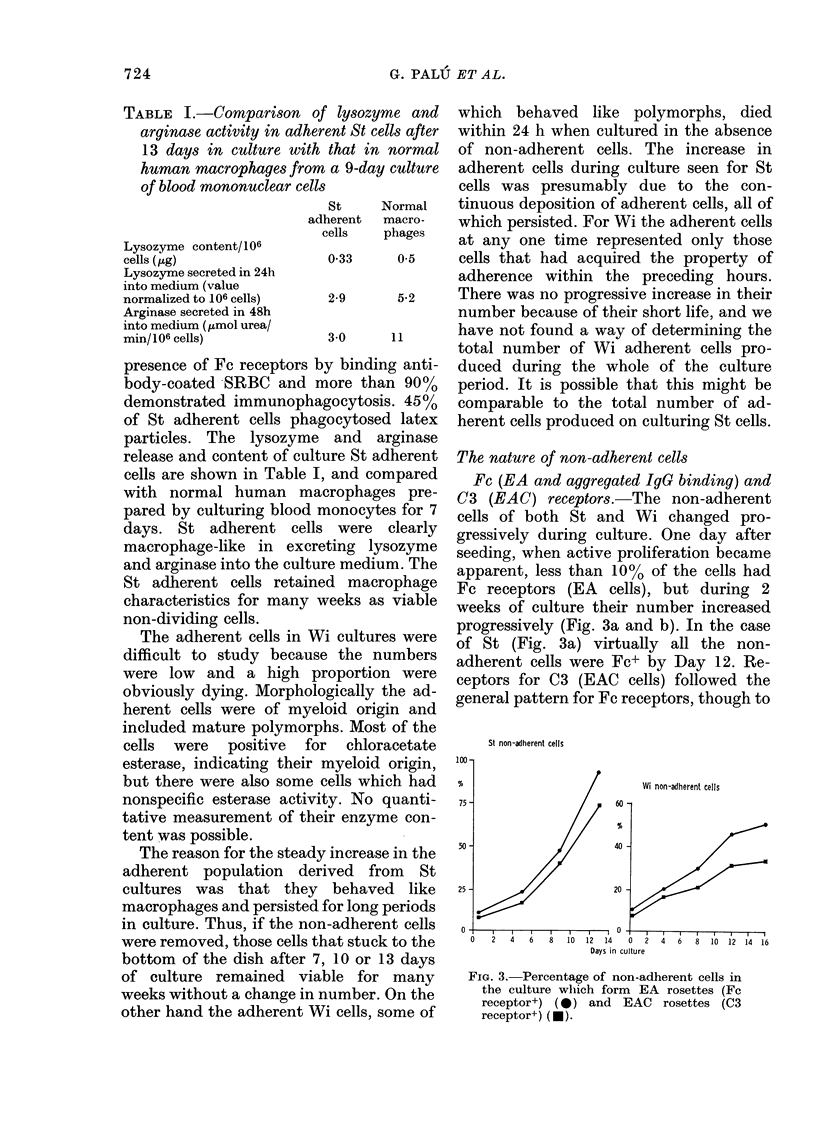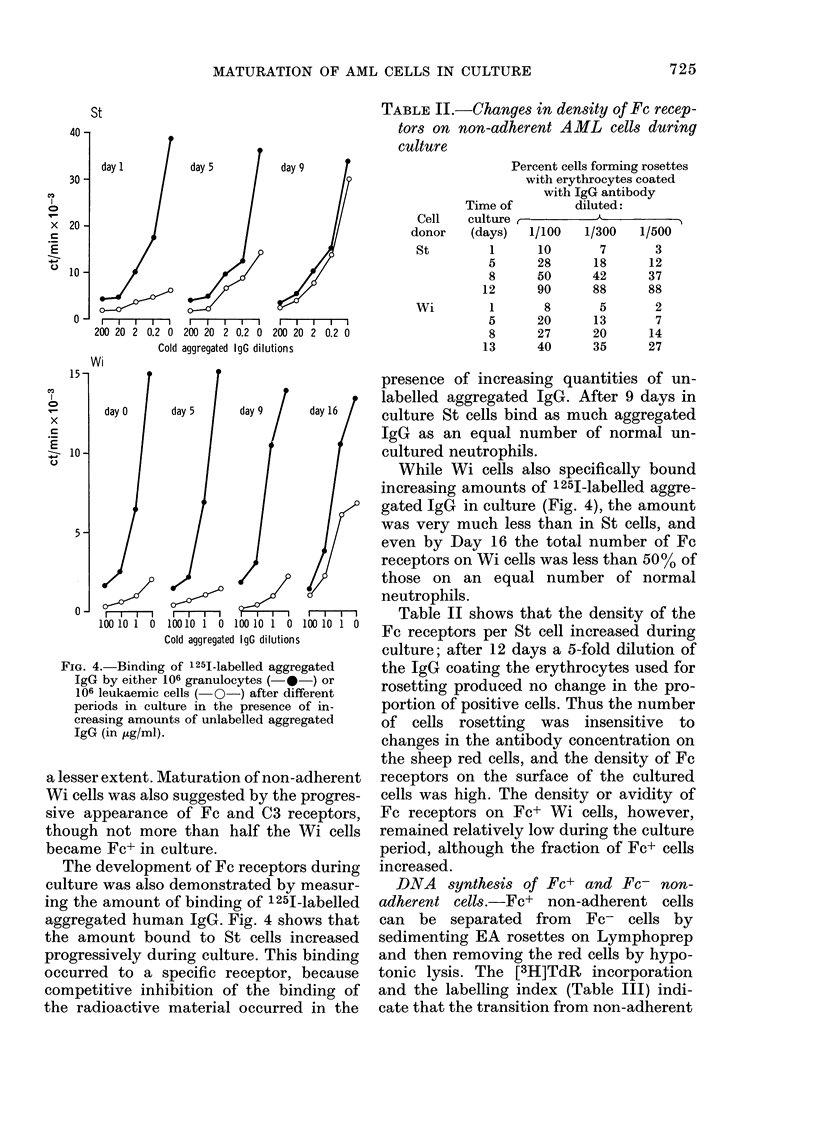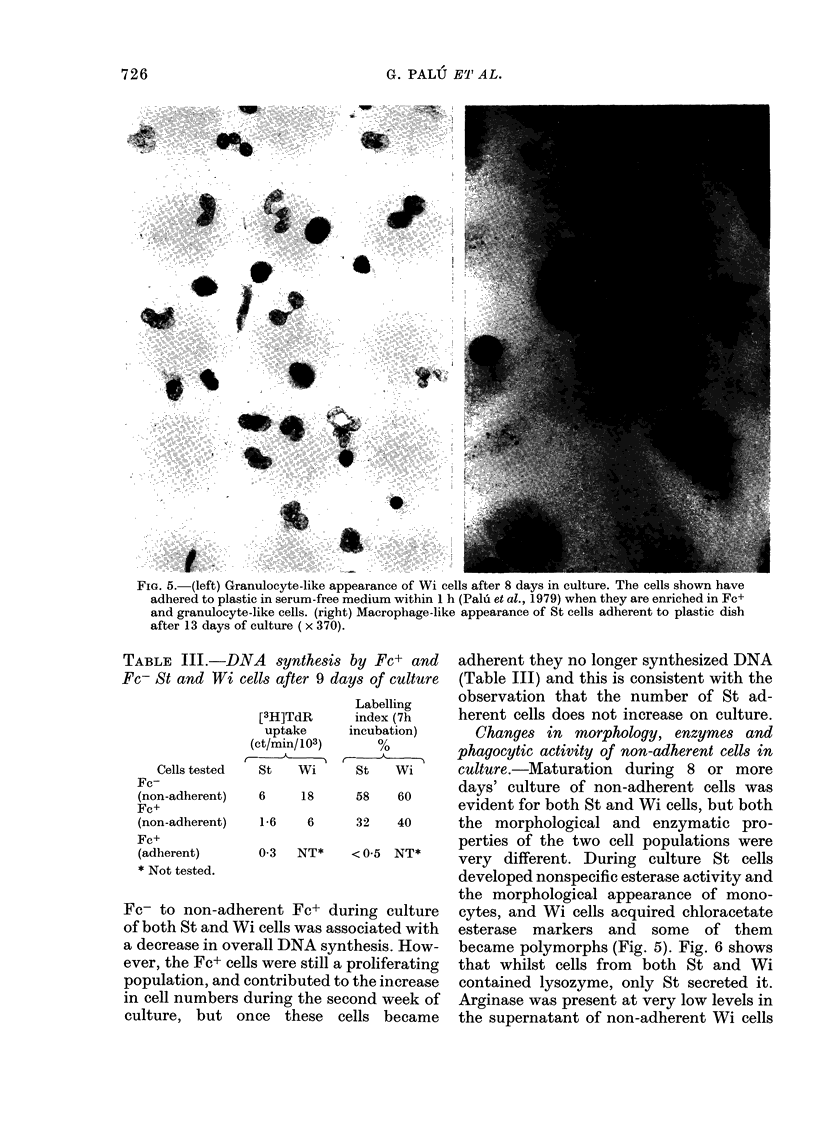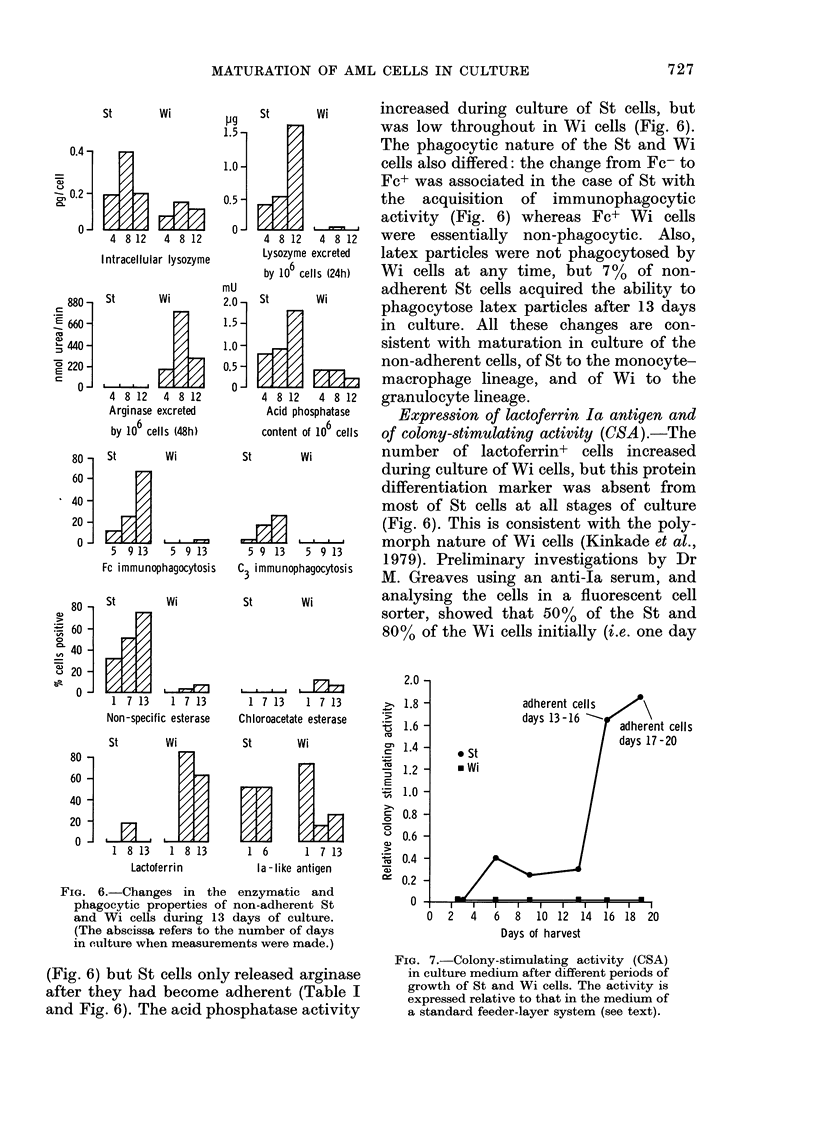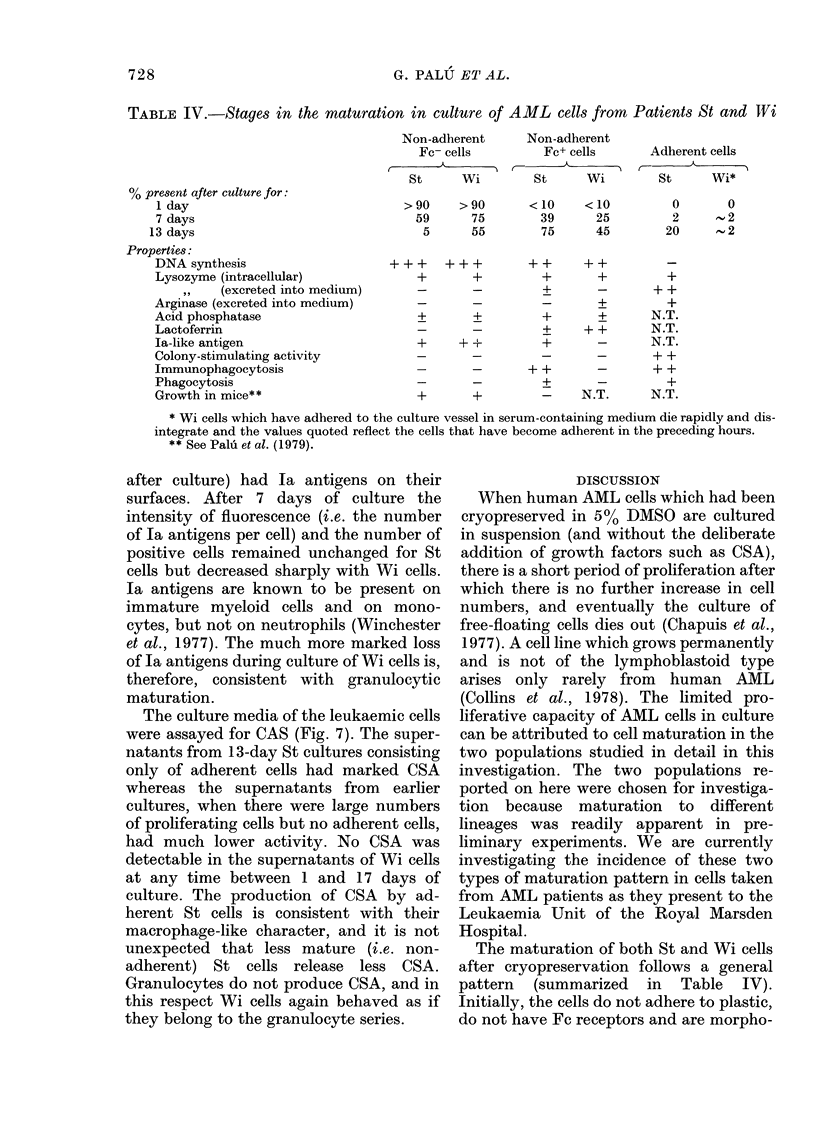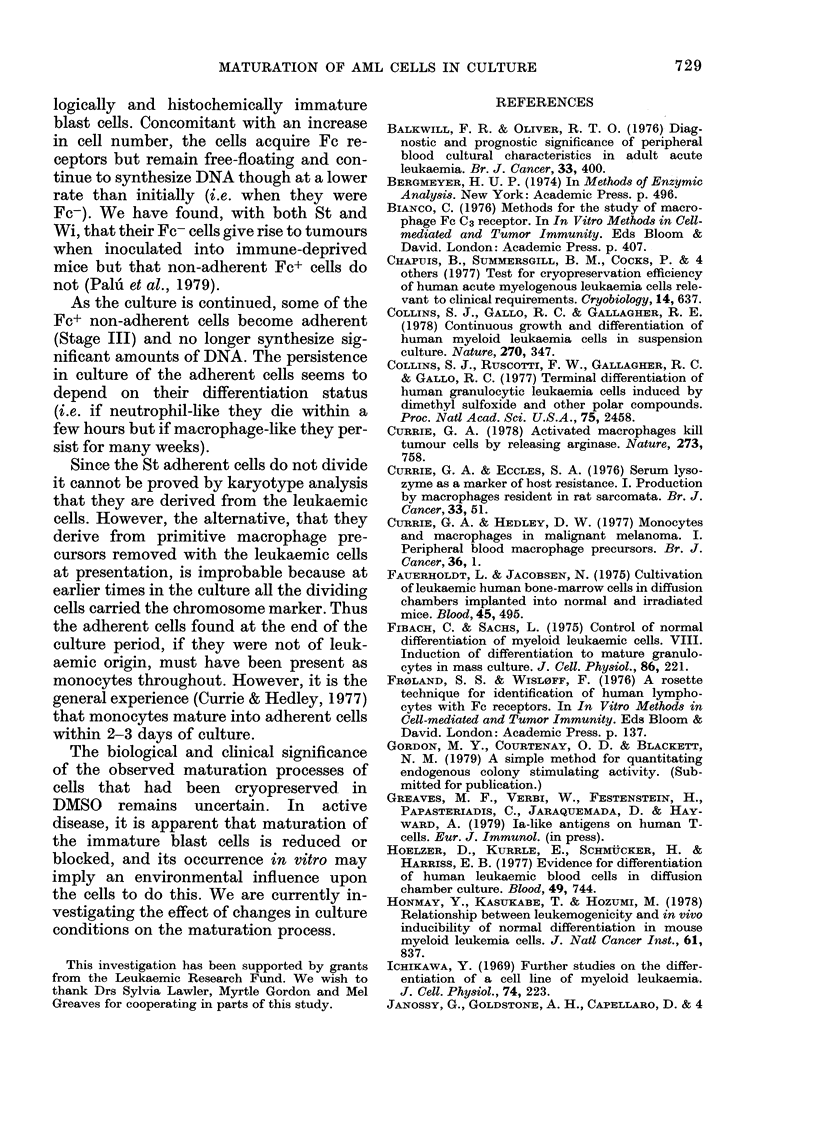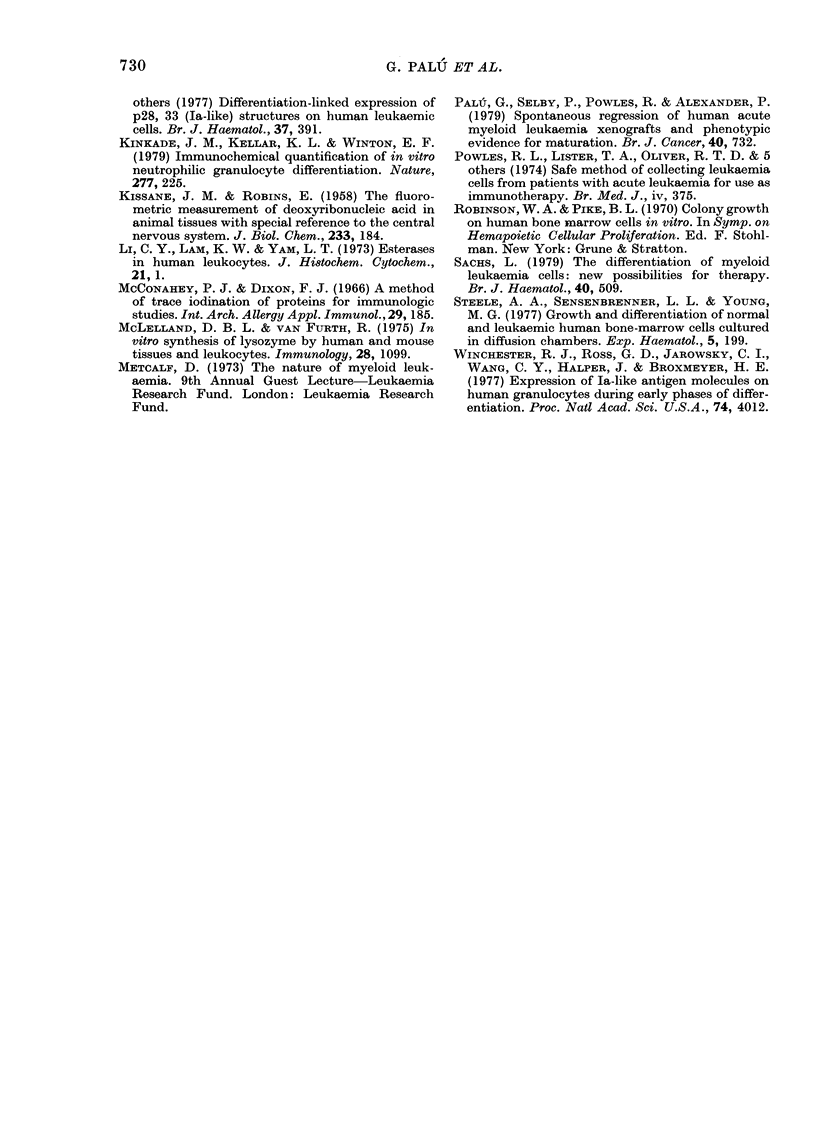# Patterns of maturation in short-term culture of human acute myeloid leukaemic cells.

**DOI:** 10.1038/bjc.1979.252

**Published:** 1979-11

**Authors:** G. Palú, R. Powles, P. Selby, B. M. Summersgill, P. Alexander

## Abstract

**Images:**


Br. J. Cancer (1979) **40**, 719

### PATTERNS OF MATURATION IN SHORT-TERM CULTURE OF HUMAN ACUTE MYELOID LEUKAEMIC CELLS

G. PALÚ, R. POWLES, P. SELBY, B. M. SUMMERSGILL AND P. ALEXANDER*

*From the Institute of Cancer Research and Royal Marsden Hospital, Sutton, Surrey*

Received 9 April 1979 Accepted 2 August 1979

**Summary.—Leukaemic cells taken from the blood of patients with acute myelogenous leukaemia (AML) frequently proliferate in suspension culture without the addition of growth factors for a limited period only. After a 6–10-fold increase in total cells, cell numbers remain constant for a time and finally decline. The main cause for this limited growth *in vitro* is not, initially at least, cell death leading to a steady state, but maturation associated in its final stages with cessation of DNA synthesis. Two populations of AML cells from Patients St and Wi respectively were studied, and progressive maturation towards mature leucocytes was demonstrated by the gradual acquisition in culture by the growing blast cells of intracellular enzymes (lysozyme, arginase, acid phosphatase and esterase being measured), surface markers (Fc and C3 receptors), of lactoferrin by Wi cells and of colony-stimulating activity by St cells, as well as changes in Ia antigens, phagocytic properties, morphology and adhesiveness to plastic. With St cells, which carried a characteristic chromosome marker, maturation terminated in cells with the characteristic properties of macrophages. At an intermediate stage, non-adherent and still-dividing St cells acquired Fc and C3 receptors and enzymes characteristic of monocytes. Wi cells progressively became neutrophil-like, and again there was an intermediate population of dividing cells which had Fc and C3 receptors and proteins such as lactoferrin and esterases, characteristic of neutrophils.**

THE CONCEPT that leukaemic blast cells from patients with acute myeloid leukaemia (AML) have the capacity to mature with loss of malignant potential has been widely discussed (Metcalf, 1973; Sachs, 1979) and examples of such a phenomenon have been demonstrated with some transplantable murine leukaemias (Ichikawa, 1969; Honmay *et al.*, 1978; Fibach & Sachs, 1975). Collins *et al.* (1977, 1978) established a progressively growing cell line from the blood of a patient with acute promyelocytic leukaemia which matured to granulocytes *in vitro*. AML cells taken directly from the patient may differentiate when maintained in Millipore chambers in mice, although claims are conflicting (Fauerholdt & Jacobsen, 1975; Hoelzer *et al.*, 1977; Steele *et al.*, 1977).

In this laboratory, Chapuis *et al.* (1977) showed that AML cells, taken from patients at first presentation using a blood–cell separator and stored in liquid $N_2$, could be grown with maintenance of the karyotype of the original leukaemia using the culture conditions described by Balkwill & Oliver (1976). Almost invariably these cultures died out after 2–4 weeks, but during this period some of the cells became more mature. In this paper we report a detailed study using physiological and biochemical markers of the *in vitro* maturation of AML cells taken from the blood of patient (St) where macrophage characteristics developed, and also of cells from a patient (Wi) which acquired polymorph characteristics *in vitro*. Cells from these two patients were chosen because both populations proliferated. Morphologically their maturation

* To whom correspondence should be addressed.

patterns were very different, and one of the populations carried a characteristic karyotype marker indicating it was the leukaemic population which was being studied.

### METHODS

### *Source of cells*

St cells were taken from a 17-year-old female with $96 \times 10^9$/l leucocytes in the blood at presentation. (As the patient died without attaining remission, the karyotype of her normal cells could not be examined, and we cannot, therefore, exclude definitely the remote possibility that the karyotypic abnormality of the leukaemic cells is constitutional.) Wi cells were from a 55-year-old male with a presentation WBC count of $26 \times 10^9$/l. Both patients had acute myelogenous leukaemia (AML) and neither patient had received any cytotoxic chemotherapy before the collection of their circulating AML cells.

The cells were collected using an NCI/IBM continuous-flow blood-cell separator (Powles *et al.*, 1974) into 70 ml of RPMI 1640 (Gibco/Bio-Cult Ltd) with preservative-free heparin (20 u/ml, final concentration) in plastic (Fenwall) blood-transfusion packs. Aliquots of $10^9$ cells were transferred to 2ml glass ampoules and frozen at a rate of $-1$°C/min in culture medium TC199 (Wellcome) with antibiotics, 5% dimethylsulphoxide (DMSO) and 25% autologous plasma (Chapuis *et al.*, 1977). The ampoules were stored in the gas phase over liquid $N_2$ at below $-150$°C.

### *Recovery of cryopreserved AML cells*

The culture medium used throughout, unless otherwise stated, was: Medium RPMI 1640 with 25mM Hepes with L-glutamine (Gibco/Bio-Cult Ltd) 100 u/ml penicillin, 100 μg/ml streptomycin, 600 mg/l additional glutamine and 15% (v/v) foetal calf serum (FCS) (Gibco Bio-Cult). Ampoules of AML cells were thawed rapidly in a 37°C water bath and diluted dropwise with 20 ml of culture medium, with continuous shaking at room temperature. After centrifugation at 400 *g* for 3 min, the pellet of cells was suspended in 10 ml of the culture medium and layered on to an equal volume of a sodium metrizoate–Ficoll mixture (Lymphoprep, Nyegaard & Co.) density 1·077 g/ml. The cell suspension was separated at 400 g at the interface for 15 min. The leucocytes lighter than 1·077 g/ml were collected at the interface and washed twice with the culture medium, and the viable cells counted by trypan-blue exclusion.

### *Technique for proliferative cultures of AML cells*

Cultures were established in 35mm Petri dishes (Corning 2.500) at a concentration of $10^6$ "live" cells in 3 ml of the tissue-culture medium. The cultures were incubated at 37°C in a moist atmosphere containing 5% $CO_2$. The culture medium was changed when it became acid, usually every 2–3 days: 1 ml of tissue-culture fluid was removed by gentle aspiration and replaced with the same volume of fresh culture medium. Cells were not removed by this process, since they had settled to the bottom of the plate.

### *Estimation of proliferation*

*Cell numbers.*—In cultures of the 2 AML cell populations, 2 types of cell occurred: those adhering firmly to the plastic of the culture containers, and non-adherent cells. The non-adherent cells were counted with a standard haemacytometer with trypan blue added to identify viable cells. The number of adherent cells was estimated both by counting the number of cells seen in the field of a calibrated objective and by lysing their cytoplasma by incubating cells for 30 min with 0·1M citric acid and 1:2000 crystal violet (Currie & Hedley, 1977) and counting the stained nuclei in a haemacytometer.

*Total DNA content and synthesis.*—(i) The DNA content of the cell cultures was measured by the fluorometric method of Kissane & Robins (1958) with minor modifications. Cells were solubilized with 0·1N NaOH and the DNA precipitated by adding 20% trichloracetic acid (TCA) at 4°C for 12 h and then centrifuging at 3500 rev/min for 7 min. The pellet was washed rapidly with 0·1mM potassium acetate at 4°C and with absolute ethanol at 60°C for 20 min in order to extract extraneous lipids. Five μl of fresh 3,5-diaminobenzoic acid dihydrochloride (DABA) in 4N HCl, decolourized with charcoal, was added to the dried pellet, and the fluorescent product of DNA was produced by heating at 60°C for 30 min. The pellet was dissolved in

150 $\mu$l of 0·6mM perchloric acid and the fluorescence produced was measured in an Aminco–Bowman double monochromator fluorometer, with an excitation wave-length of 420 nm and emission of 520 nm. Highly polymerized calf thymus DNA (BDH Chemicals) in 0·1N NaOH was used as the standard.

(ii) DNA synthesis is the adherent and non-adherent cells was measured using the incorporating of [$^3$H]TdR. One $\times 10^6$ non-adherent cells were suspended in 2·7 ml of fresh culture medium, in a 35mm Petri dish, and 0·3 ml of 10 $\mu$Ci/ml (370 kBq/ml) [$^3$H]TdR (Amersham) sp. act. 20–30 ci/mM (740–1110 GBq/mM) was added. After 3h incubation at 37°C the cells were collected on a 0·22$\mu$m Millipore filter. Any cells which had become adherent to the dish during the 1h [$^3$H]TdR pulse were solubilized in 1 ml of 1% solution of Sarkosyl (NL-97, Ciba-Geigy) in 0·5N NaOH for 10–20 min before being passed through the filter after precipitation with 10% TCA. $^3$H-labelled DNA was measured with a Packard Beta Scintillation counter.

Estimation of DNA synthesis in cells which were adherent during proliferative culture was also estimated by this alkaline-sarcosine technique.

Autoradiographs of adherent and non-adherent cells labelled with [$^3$H]TdR were prepared by a conventional dipping technique.

*Maturation studies*

*Morphology and cytochemistry.*—Slides of the cultured cells were fixed and stained with Geimsa, and for the enzymes nonspecific esterase (NSE) and chloracetate esterase to show monocyte and polymorph differentiation (Li *et al.*, 1973). Adherent cells were fixed and stained directly on the culture dishes.

*Fc receptors.*—(i) EA rosettes: Non-adherent cells were washed and $10^6$ cells were resuspended in 1 ml of culture medium without FCS. Four $\times 10^7$ sheep erythrocytes coated with the IgG fraction of a rabbit anti-sheep-erythrocyte serum (kindly supplied by Dr G. Currie) were added. The percentage of rosette-forming cells was estimated by the technique of Frøland & Wisløff (1976). The cell suspensions were centrifuged at a very low *g* for 10–20 min at room temperature, resuspended, and counted in a haemacytometer under phase contrast. Leucocytes with 3 or more attached erythrocytes, or those which had phagocytosed erythrocytes, were scored as positive. Uncoated sheep erythrocytes were used as a control. Adherent cells tended to phagocytose the erythrocytes rather than binding them to form the rosettes, so positive cells were determined mainly by erythrophagocytosis. Different dilutions of the same IgG anti-SRBC antibody were used to study Fc density.

(ii) Separation of $Fc^+$ and $Fc^-$ non-adherent cells: To study the DNA synthetic activity of $Fc^+$ and $Fc^-$ cell populations, non-adherent cells were labelled with [$^3$H]TdR for 1–7 h and $Fc^+$ separated from $Fc^-$ by sedimenting the EA-rosetting cells on Lymphoprep. After removing the red cells by hypotonic lysis, the radioactivity was measured in both fractions, and autoradiographs were performed in the usual way.

(iii) Density of Fc receptors: When the number of Fc receptors per cell is relatively low, higher concentrations of antibody-coated red cells are required to form rosettes than when more Fc receptors are present. Changes in the density of Fc receptors can, therefore, be detected by measuring changes in the fraction of cells which form rosettes when the red cells are coated with progressively decreasing amounts of antibody. If the density of Fc receptors is high, the number of rosettes will be insensitive to changes in the antibody on the EA cells, whereas with low densities of Fc receptors rosetting increases with increasing antibody concentration.

(iv) Aggregated IgG binding: Non-adherent cells were incubated at 4°C for 1 h with $I^{125}$-labelled aggregated human IgG. After centrifugation and 3 washings, the amount of isotope bound was measured by scintillation counting. The radiolabelled aggregated IgG was produced using a Cohn Fraction II human IgG (Miles Laboratories). Aggregation was obtained by heating a 20mg/ml solution in PBS (pH 7·2) at 63°C for 30 min. After centrifugation at 2000 rev/min for 10 min the supernatant was passed through a Sephadex G200 column and the fraction collected with the void volume and concentrated with an Amicon filter (cut-off 10,000 daltons). The chloramine T method (McConahey & Dixon, 1966) was used to label the aggregated IgG. Controls included the uptake of radiolabelled aggregated IgG by normal human granulocytes, and the specificity of the binding was checked by blocking the uptake of the labelled aggregated IgG with cold aggregated IgG.

(v) $C_3$ receptors (EAC rosette technique): $C_3$ receptors on the surfaces of the cultured cells were detected by the technique described by Bianco (1976) with minor modifications. Non-adherent cells were resuspended in 1 ml of medium without FCS and $4 \times 10^7$ sheep erythrocytes coated with rabbit anti-sheep IgM plus complement were added. The antiserum was produced by i.v. injection of 10% (v/v) washed sheep erythrocytes into a rabbit (white N.Z.). Serum was collected 7–10 days later and the euglobulin precipitated by extensive dialysis with distilled water and separation by centrifugation. The product was then redissolved in G200 buffer (PBS pH 7·2 with 0·5M NaCl) and fractionated in a G200 column. The IgM in the ascending peak in the void volume was collected and concentrated using an Amicon filter as before. The sheep red cells (E) were coated with an appropriate solution of IgM anti-E after incubation at 37°C for 30 min with continuous shaking. The coated erythrocytes (EM) were washed twice at room temperature with medium, and complement (fresh DBA2 serum 1 : 5 diluted) was added by incubation for 30 min at 37°C in the presence of gelatin Veronal buffer (Bianco, 1976). Rosetting and phagocytosis were assessed as described above (EA-rosette test). Specific IgM-coated sheep erythrocytes without complement were used as a control.

(vi) Immunophagocytosis: This was determined by counting the number of cells that phagocytosed IgG- and IgM/complement-coated sheep erythrocytes. Erythrocytes which formed rosettes were removed by hypotonic lysis and the cells which phagocytosed erythrocytes were estimated as described above.

(vii) Latex phagocytosis: Latex particles (Sigma Ltd, size 0·79 $\mu$m) at a concentration of $6 \times 10^8$/ml, to give an approximate ratio of 100 particles/cell, were incubated at 37°C for 60 min with cultured cells. Cells were then washed thoroughly, fixed and stained with Geimsa, and examined under a phase-contrast microscope.

*Enzyme markers*.—(i) Lysozyme: Neutrophils contain but do not release intracellular lysozyme, whereas macrophages synthesize and actually excrete this enzyme (and arginase) into the medium (McLelland & van Furth, 1975). $10^6$ non-adherent cells, and dishes with only adherent cells, were incubated at fresh medium for 24 h at 37°C. Supernatants from these cultures were tested for lysozyme as described by Currie & Eccles (1976). Intracellular contents of lysozyme was assayed by lysing the cells by freezing and thawing $\times 3$.

(ii) Arginase: Supernatants from cultures of adherent and non-adherent cells were tested for arginase activity as described by Currie (1978).

(iii) Acid phosphatase: Cultured cells were lysed by 1ml saline containing 0·1% triton X-100 (v/v) and after centrifugation acid-phosphatase activity was assessed on the lysate as described by Bergmeyer (1974).

*Ia-like surface markers*.—A chicken antiserum with specificity for human p28/33 antigens raised against B-cell "Ia" DR antigens as described by Greaves *et al.* (1979) was used. Non-adherent cells at various times of culture were studied for their ability to express this surface marker. Cells were examined by Dr M. Greaves for membrane fluorescence with the antiserum, using both a fluorescence microscope and a fluorescence-activated cell sorter (FACS), as described by Janossy *et al.* (1977).

*Lactoferrin*.—Cells were fixed for 10 min in acetone at room temperature and incubated with a purified rabbit anti-human-lactoferrin serum (Behringwerke) followed by a fluorescein-labelled goat anti-rabbit serum (Miles) for 30 min at 37°C. Slides were viewed using a Zeiss photomicroscope 3, equipped for fluorescence with a caesium iodine lamp. Purified human neutrophils served as a positive control.

*Estimation of colony-stimulating activity (CSA)*.—Cultures were seeded with $10^6$ cells and at various times afterwards supernatant medium (taken over a 3-day period) was analysed for CSA activity. St adherent cells (between $7 \times 10^5$ and $1 \times 10^6$ cells per dish) were also assessed for CSA production. These media were incorporated into soft-agar cultures of normal human marrow cells, without feeder layers or other source of CSA. The number of colonies stimulated by the conditioned media was compared with the number of colonies stimulated by feeder layers of human mononuclear cells and rat haemolysate (Robinson & Pike, 1970; Gordon *et al.*, 1979) to obtain a relative measure of CSA.

*Karyotypes of cells in culture*.—Performed as described by Chapuis *et al.* (1977).

### RESULTS

### *Cell multiplication and DNA synthesis*

*DNA synthesis.*—Cultures of both populations of cells contained two cell types: those adhering firmly to the plastic, and non-adherent cells. In cultures of St cells the adherent population increases progressively (Fig. 1) and after 2 weeks constituted $\sim 20\%$ of all cells. With Wi cells

FIG. 1.—Changes in cell numbers and DNA content of St and Wi leukaemia cells in cultures of 3 ml with an initial inoculum of $10^6$ cells. —■— Number of non-adherent cells $\times 10^5$; —●— $\mu$g DNA in all the non-adherent cells. —□— Number of adherent cells $\times 10^5$; —○— $\mu$g DNA in all the adherent cells.

the adherent population at any one time was small and remained relatively constant after the third day of culture, and then rarely exceeded $10^5$ cells per dish. Fig. 1 indicates that for both St and Wi cells some $20\%$ of the non-adherent cells disappeared during the first day of culture. This was probably due to damage not reflected by gross leakage, since all cells excluded trypan blue and sedimented to the interface in a Lymphoprep separation. The total number of cells increased progressively 6–8-fold during the next 10 days, but thereafter the number slowly declined in spite of medium change. The total DNA content of the cultures (Fig. 1) closely followed the cell count, showing

FIG. 2.—DNA synthesis by St and Wi cells in culture. —◆— Number of non-adherent cells $\times 10^{-6}$/3 ml of culture. —●— ct/min $\times 10^4$ per $10^3$ cells after 3h incubation in [$^3$H]TdR. —■— Labelling index ($\times 10$) after 3h incubation in [$^3$H]TdR.

that in the plateau period the cells did not continue to synthesize DNA. Indeed, the DNA content per cell was slightly less for cultures in the plateau than in the logarithmic phase.

Both procedures used to determine DNA synthesis of cultured (St) and (Wi) cells showed high activity early in the culture (4th day) which declined slowly during the next 6 days and then fell to very low levels (Fig. 2a and b), indicating that the cause of the plateau of cell numbers in the culture was a reduction in proliferation rate and not increased cell loss balancing proliferation.

*Karyotype studies.*—At the time of diagnosis all the metaphase cells in the marrow of Patient St had additional material in the long arm of Chromosome 11. Chromosome analysis was also performed on St cells after they had been in proliferation culture for 8 days. Fifty cells at metaphase were analysed, and all had 46 chromosomes and carried the change in the Chromosome 11 seen at presentation.

### *The nature of adherent cells*

St adherent cells were mononuclear (see Fig. 7) and had the histochemical characteristics of macrophages (nonspecific-esterase-positive, chloracetate-esterase-negative). All St adherent cells showed the

TABLE I.—*Comparison of lysozyme and arginase activity in adherent St cells after 13 days in culture with that in normal human macrophages from a 9-day culture of blood mononuclear cells*

| | St adherent cells | Normal macrophages |
|---|---|---|
| Lysozyme content/$10^6$ cells ($\mu$g) | 0·33 | 0·5 |
| Lysozyme secreted in 24h into medium (value normalized to $10^6$ cells) | 2·9 | 5·2 |
| Arginase secreted in 48h into medium ($\mu$mol urea/ min/$10^6$ cells) | 3·0 | 11 |

presence of Fc receptors by binding antibody-coated SRBC and more than 90% demonstrated immunophagocytosis. 45% of St adherent cells phagocytosed latex particles. The lysozyme and arginase release and content of culture St adherent cells are shown in Table I, and compared with normal human macrophages prepared by culturing blood monocytes for 7 days. St adherent cells were clearly macrophage-like in excreting lysozyme and arginase into the culture medium. The St adherent cells retained macrophage characteristics for many weeks as viable non-dividing cells.

The adherent cells in Wi cultures were difficult to study because the numbers were low and a high proportion were obviously dying. Morphologically the adherent cells were of myeloid origin and included mature polymorphs. Most of the cells were positive for chloracetate esterase, indicating their myeloid origin, but there were also some cells which had nonspecific esterase activity. No quantitative measurement of their enzyme content was possible.

The reason for the steady increase in the adherent population derived from St cultures was that they behaved like macrophages and persisted for long periods in culture. Thus, if the non-adherent cells were removed, those cells that stuck to the bottom of the dish after 7, 10 or 13 days of culture remained viable for many weeks without a change in number. On the other hand the adherent Wi cells, some of which behaved like polymorphs, died within 24 h when cultured in the absence of non-adherent cells. The increase in adherent cells during culture seen for St cells was presumably due to the continuous deposition of adherent cells, all of which persisted. For Wi the adherent cells at any one time represented only those cells that had acquired the property of adherence within the preceding hours. There was no progressive increase in their number because of their short life, and we have not found a way of determining the total number of Wi adherent cells produced during the whole of the culture period. It is possible that this might be comparable to the total number of adherent cells produced on culturing St cells.

*The nature of non-adherent cells*

*Fc (EA and aggregated IgG binding) and C3 (EAC) receptors.*—The non-adherent cells of both St and Wi changed progressively during culture. One day after seeding, when active proliferation became apparent, less than 10% of the cells had Fc receptors (EA cells), but during 2 weeks of culture their number increased progressively (Fig. 3a and b). In the case of St (Fig. 3a) virtually all the non-adherent cells were $Fc^+$ by Day 12. Receptors for C3 (EAC cells) followed the general pattern for Fc receptors, though to

FIG. 3.—Percentage of non-adherent cells in the culture which form EA rosettes (Fc receptor$^+$) (●) and EAC rosettes (C3 receptor$^+$) (■).

FIG. 4.—Binding of $^{125}$I-labelled aggregated IgG by either $10^6$ granulocytes (—●—) or $10^6$ leukaemic cells (—○—) after different periods in culture in the presence of increasing amounts of unlabelled aggregated IgG (in $\mu$g/ml).

a lesser extent. Maturation of non-adherent Wi cells was also suggested by the progressive appearance of Fc and C3 receptors, though not more than half the Wi cells became Fc+ in culture.

The development of Fc receptors during culture was also demonstrated by measuring the amount of binding of $^{125}$I-labelled aggregated human IgG. Fig. 4 shows that the amount bound to St cells increased progressively during culture. This binding occurred to a specific receptor, because competitive inhibition of the binding of the radioactive material occurred in the

TABLE II.—*Changes in density of Fc receptors on non-adherent AML cells during culture*

| Cell donor | Time of culture (days) | Percent cells forming rosettes with erythrocytes coated with IgG antibody diluted: 1/100 | 1/300 | 1/500 |
|---|---|---|---|---|
| St | 1 | 10 | 7 | 3 |
| | 5 | 28 | 18 | 12 |
| | 8 | 50 | 42 | 37 |
| | 12 | 90 | 88 | 88 |
| Wi | 1 | 8 | 5 | 2 |
| | 5 | 20 | 13 | 7 |
| | 8 | 27 | 20 | 14 |
| | 13 | 40 | 35 | 27 |

presence of increasing quantities of unlabelled aggregated IgG. After 9 days in culture St cells bind as much aggregated IgG as an equal number of normal uncultured neutrophils.

While Wi cells also specifically bound increasing amounts of $^{125}$I-labelled aggregated IgG in culture (Fig. 4), the amount was very much less than in St cells, and even by Day 16 the total number of Fc receptors on Wi cells was less than 50% of those on an equal number of normal neutrophils.

Table II shows that the density of the Fc receptors per St cell increased during culture; after 12 days a 5-fold dilution of the IgG coating the erythrocytes used for rosetting produced no change in the proportion of positive cells. Thus the number of cells rosetting was insensitive to changes in the antibody concentration on the sheep red cells, and the density of Fc receptors on the surface of the cultured cells was high. The density or avidity of Fc receptors on Fc+ Wi cells, however, remained relatively low during the culture period, although the fraction of Fc+ cells increased.

*DNA synthesis of Fc+ and Fc− non-adherent cells.*—Fc+ non-adherent cells can be separated from Fc− cells by sedimenting EA rosettes on Lymphoprep and then removing the red cells by hypotonic lysis. The [$^3$H]TdR incorporation and the labelling index (Table III) indicate that the transition from non-adherent

FIG. 5.—(left) Granulocyte-like appearance of Wi cells after 8 days in culture. The cells shown have adhered to plastic in serum-free medium within 1 h (Palú *et al.*, 1979) when they are enriched in Fc+ and granulocyte-like cells. (right) Macrophage-like appearance of St cells adherent to plastic dish after 13 days of culture ( × 370).

TABLE III.—*DNA synthesis by Fc+ and Fc− St and Wi cells after* 9 *days of culture*

| | [$^3$H]TdR uptake (ct/min/$10^3$) | | Labelling index (7h incubation) % | |
|---|---|---|---|---|
| Cells tested | St | Wi | St | Wi |
| Fc− (non-adherent) | 6 | 18 | 58 | 60 |
| Fc+ (non-adherent) | 1·6 | 6 | 32 | 40 |
| Fc+ (adherent) | 0·3 | NT* | < 0·5 | NT* |

* Not tested.

Fc− to non-adherent Fc+ during culture of both St and Wi cells was associated with a decrease in overall DNA synthesis. However, the Fc+ cells were still a proliferating population, and contributed to the increase in cell numbers during the second week of culture, but once these cells became adherent they no longer synthesized DNA (Table III) and this is consistent with the observation that the number of St adherent cells does not increase on culture.

*Changes in morphology, enzymes and phagocytic activity of non-adherent cells in culture.*—Maturation during 8 or more days' culture of non-adherent cells was evident for both St and Wi cells, but both the morphological and enzymatic properties of the two cell populations were very different. During culture St cells developed nonspecific esterase activity and the morphological appearance of monocytes, and Wi cells acquired chloracetate esterase markers and some of them became polymorphs (Fig. 5). Fig. 6 shows that whilst cells from both St and Wi contained lysozyme, only St secreted it. Arginase was present at very low levels in the supernatant of non-adherent Wi cells

FIG. 6.—Changes in the enzymatic and phagocytic properties of non-adherent St and Wi cells during 13 days of culture. (The abscissa refers to the number of days in culture when measurements were made.)

(Fig. 6) but St cells only released arginase after they had become adherent (Table I and Fig. 6). The acid phosphatase activity increased during culture of St cells, but was low throughout in Wi cells (Fig. 6). The phagocytic nature of the St and Wi cells also differed: the change from $Fc^-$ to $Fc^+$ was associated in the case of St with the acquisition of immunophagocytic activity (Fig. 6) whereas $Fc^+$ Wi cells were essentially non-phagocytic. Also, latex particles were not phagocytosed by Wi cells at any time, but 7% of non-adherent St cells acquired the ability to phagocytose latex particles after 13 days in culture. All these changes are consistent with maturation in culture of the non-adherent cells, of St to the monocyte–macrophage lineage, and of Wi to the granulocyte lineage.

*Expression of lactoferrin Ia antigen and of colony-stimulating activity (CSA).*—The number of $lactoferrin^+$ cells increased during culture of Wi cells, but this protein differentiation marker was absent from most of St cells at all stages of culture (Fig. 6). This is consistent with the polymorph nature of Wi cells (Kinkade *et al.*, 1979). Preliminary investigations by Dr M. Greaves using an anti-Ia serum, and analysing the cells in a fluorescent cell sorter, showed that 50% of the St and 80% of the Wi cells initially (*i.e.* one day

FIG. 7.—Colony-stimulating activity (CSA) in culture medium after different periods of growth of St and Wi cells. The activity is expressed relative to that in the medium of a standard feeder-layer system (see text).

TABLE IV.—*Stages in the maturation in culture of AML cells from Patients St and Wi*

| | Non-adherent Fc⁻ cells | | Non-adherent Fc⁺ cells | | Adherent cells | |
|---|---|---|---|---|---|---|
| | St | Wi | St | Wi | St | Wi* |
| *% present after culture for:* | | | | | | |
| 1 day | > 90 | > 90 | < 10 | < 10 | 0 | 0 |
| 7 days | 59 | 75 | 39 | 25 | 2 | ~2 |
| 13 days | 5 | 55 | 75 | 45 | 20 | ~2 |
| *Properties:* | | | | | | |
| DNA synthesis | +++ | +++ | ++ | ++ | − | |
| Lysozyme (intracellular) | + | + | + | + | + | |
| ,, (excreted into medium) | − | − | ± | − | ++ | |
| Arginase (excreted into medium) | − | − | − | ± | + | |
| Acid phosphatase | ± | ± | + | ± | N.T. | |
| Lactoferrin | − | − | ± | ++ | N.T. | |
| Ia-like antigen | + | ++ | + | − | N.T. | |
| Colony-stimulating activity | − | − | − | − | ++ | |
| Immunophagocytosis | − | − | ++ | − | ++ | |
| Phagocytosis | − | − | ± | − | + | |
| Growth in mice** | + | + | − | N.T. | N.T. | |

* Wi cells which have adhered to the culture vessel in serum-containing medium die rapidly and disintegrate and the values quoted reflect the cells that have become adherent in the preceding hours.
** See Palú *et al.* (1979).

after culture) had Ia antigens on their surfaces. After 7 days of culture the intensity of fluorescence (*i.e.* the number of Ia antigens per cell) and the number of positive cells remained unchanged for St cells but decreased sharply with Wi cells. Ia antigens are known to be present on immature myeloid cells and on monocytes, but not on neutrophils (Winchester *et al.*, 1977). The much more marked loss of Ia antigens during culture of Wi cells is, therefore, consistent with granulocytic maturation.

The culture media of the leukaemic cells were assayed for CAS (Fig. 7). The supernatants from 13-day St cultures consisting only of adherent cells had marked CSA whereas the supernatants from earlier cultures, when there were large numbers of proliferating cells but no adherent cells, had much lower activity. No CSA was detectable in the supernatants of Wi cells at any time between 1 and 17 days of culture. The production of CSA by adherent St cells is consistent with their macrophage-like character, and it is not unexpected that less mature (*i.e.* non-adherent) St cells release less CSA. Granulocytes do not produce CSA, and in this respect Wi cells again behaved as if they belong to the granulocyte series.

### DISCUSSION

When human AML cells which had been cryopreserved in 5% DMSO are cultured in suspension (and without the deliberate addition of growth factors such as CSA), there is a short period of proliferation after which there is no further increase in cell numbers, and eventually the culture of free-floating cells dies out (Chapuis *et al.*, 1977). A cell line which grows permanently and is not of the lymphoblastoid type arises only rarely from human AML (Collins *et al.*, 1978). The limited proliferative capacity of AML cells in culture can be attributed to cell maturation in the two populations studied in detail in this investigation. The two populations reported on here were chosen for investigation because maturation to different lineages was readily apparent in preliminary experiments. We are currently investigating the incidence of these two types of maturation pattern in cells taken from AML patients as they present to the Leukaemia Unit of the Royal Marsden Hospital.

The maturation of both St and Wi cells after cryopreservation follows a general pattern (summarized in Table IV). Initially, the cells do not adhere to plastic, do not have Fc receptors and are morphologically and histochemically immature blast cells. Concomitant with an increase in cell number, the cells acquire Fc receptors but remain free-floating and continue to synthesize DNA though at a lower rate than initially (*i.e.* when they were $Fc^-$). We have found, with both St and Wi, that their $Fc^-$ cells give rise to tumours when inoculated into immune-deprived mice but that non-adherent $Fc^+$ cells do not (Palú *et al.*, 1979).

As the culture is continued, some of the $Fc^+$ non-adherent cells become adherent (Stage III) and no longer synthesize significant amounts of DNA. The persistence in culture of the adherent cells seems to depend on their differentiation status (*i.e.* if neutrophil-like they die within a few hours but if macrophage-like they persist for many weeks).

Since the St adherent cells do not divide it cannot be proved by karyotype analysis that they are derived from the leukaemic cells. However, the alternative, that they derive from primitive macrophage precursors removed with the leukaemic cells at presentation, is improbable because at earlier times in the culture all the dividing cells carried the chromosome marker. Thus the adherent cells found at the end of the culture period, if they were not of leukaemic origin, must have been present as monocytes throughout. However, it is the general experience (Currie & Hedley, 1977) that monocytes mature into adherent cells within 2–3 days of culture.

The biological and clinical significance of the observed maturation processes of cells that had been cryopreserved in DMSO remains uncertain. In active disease, it is apparent that maturation of the immature blast cells is reduced or blocked, and its occurrence *in vitro* may imply an environmental influence upon the cells to do this. We are currently investigating the effect of changes in culture conditions on the maturation process.

This investigation has been supported by grants from the Leukaemic Research Fund. We wish to thank Drs Sylvia Lawler, Myrtle Gordon and Mel Greaves for cooperating in parts of this study.

REFERENCES

BALKWILL, F. R. & OLIVER, R. T. O. (1976) Diagnostic and prognostic significance of peripheral blood cultural characteristics in adult acute leukaemia. *Br. J. Cancer*, **33**, 400.

BERGMEYER, H. U. P. (1974) In *Methods of Enzymic Analysis*. New York: Academic Press. p. 496.

BIANCO, C. (1976) Methods for the study of macrophage Fc $C_3$ receptor. In *In Vitro Methods in Cell-mediated and Tumor Immunity*. Eds Bloom & David. London: Academic Press. p. 407.

CHAPUIS, B., SUMMERSGILL, B. M., COCKS, P. & 4 others (1977) Test for cryopreservation efficiency of human acute myelogenous leukaemia cells relevant to clinical requirements. *Cryobiology*, **14**, 637.

COLLINS, S. J., GALLO, R. C. & GALLAGHER, R. E. (1978) Continuous growth and differentiation of human myeloid leukaemia cells in suspension culture. *Nature*, **270**, 347.

COLLINS, S. J., RUSCOTTI, F. W., GALLAGHER, R. C. & GALLO, R. C. (1977) Terminal differentiation of human granulocytic leukaemia cells induced by dimethyl sulfoxide and other polar compounds. *Proc. Natl Acad. Sci. U.S.A.*, **75**, 2458.

CURRIE, G. A. (1978) Activated macrophages kill tumour cells by releasing arginase. *Nature*, **273**, 758.

CURRIE, G. A. & ECCLES, S. A. (1976) Serum lysozyme as a marker of host resistance. I. Production by macrophages resident in rat sarcomata. *Br. J. Cancer*, **33**, 51.

CURRIE, G. A. & HEDLEY, D. W. (1977) Monocytes and macrophages in malignant melanoma. I. Peripheral blood macrophage precursors. *Br. J. Cancer*, **36**, 1.

FAUERHOLDT, L. & JACOBSEN, N. (1975) Cultivation of leukaemic human bone-marrow cells in diffusion chambers implanted into normal and irradiated mice. *Blood*, **45**, 495.

FIBACH, C. & SACHS, L. (1975) Control of normal differentiation of myeloid leukaemic cells. VIII. Induction of differentiation to mature granulocytes in mass culture. *J. Cell. Physiol.*, **86**, 221.

FRØLAND, S. S. & WISLØFF, F. (1976) A rosette technique for identification of human lymphocytes with Fc receptors. In *In Vitro Methods in Cell-mediated and Tumor Immunity*. Eds Bloom & David. London: Academic Press. p. 137.

GORDON, M. Y., COURTENAY, O. D. & BLACKETT, N. M. (1979) A simple method for quantitating endogenous colony stimulating activity. (Submitted for publication.)

GREAVES, M. F., VERBI, W., FESTENSTEIN, H., PAPASTERIADIS, C., JARAQUEMADA, D. & HAYWARD, A. (1979) Ia-like antigens on human T-cells. *Eur. J. Immunol.* (in press).

HOELZER, D., KURRLE, E., SCHMÜCKER, H. & HARRISS, E. B. (1977) Evidence for differentiation of human leukaemic blood cells in diffusion chamber culture. *Blood*, **49**, 744.

HONMAY, Y., KASUKABE, T. & HOZUMI, M. (1978) Relationship between leukemogenicity and *in vivo* inducibility of normal differentiation in mouse myeloid leukemia cells. *J. Natl Cancer Inst.*, **61**, 837.

ICHIKAWA, Y. (1969) Further studies on the differentiation of a cell line of myeloid leukaemia. *J. Cell. Physiol.*, **74**, 223.

JANOSSY, G., GOLDSTONE, A. H., CAPELLARO, D. & 4

others (1977) Differentiation-linked expression of p28, 33 (Ia-like) structures on human leukaemic cells. *Br. J. Haematol.*, **37**, 391.

KINKADE, J. M., KELLAR, K. L. & WINTON, E. F. (1979) Immunochemical quantification of *in vitro* neutrophilic granulocyte differentiation. *Nature*, **277**, 225.

KISSANE, J. M. & ROBINS, E. (1958) The fluorometric measurement of deoxyribonucleic acid in animal tissues with special reference to the central nervous system. *J. Biol. Chem.*, **233**, 184.

LI, C. Y., LAM, K. W. & YAM, L. T. (1973) Esterases in human leukocytes. *J. Histochem. Cytochem.*, **21**, 1.

MCCONAHEY, P. J. & DIXON, F. J. (1966) A method of trace iodination of proteins for immunologic studies. *Int. Arch. Allergy Appl. Immunol.*, **29**, 185.

MCLELLAND, D. B. L. & VAN FURTH, R. (1975) *In vitro* synthesis of lysozyme by human and mouse tissues and leukocytes. *Immunology*, **28**, 1099.

METCALF, D. (1973) The nature of myeloid leukaemia. 9th Annual Guest Lecture—Leukaemia Research Fund. London: Leukaemia Research Fund.

PALÚ, G., SELBY, P., POWLES, R. & ALEXANDER, P. (1979) Spontaneous regression of human acute myeloid leukaemia xenografts and phenotypic evidence for maturation. *Br. J. Cancer*, **40**, 732.

POWLES, R. L., LISTER, T. A., OLIVER, R. T. D. & 5 others (1974) Safe method of collecting leukaemia cells from patients with acute leukaemia for use as immunotherapy. *Br. Med. J.*, iv, 375.

ROBINSON, W. A. & PIKE, B. L. (1970) Colony growth on human bone marrow cells *in vitro*. In *Symp. on Hemapoietic Cellular Proliferation*. Ed. F. Stohlman. New York: Grune & Stratton.

SACHS, L. (1979) The differentiation of myeloid leukaemia cells: new possibilities for therapy. *Br. J. Haematol.*, **40**, 509.

STEELE, A. A., SENSENBRENNER, L. L. & YOUNG, M. G. (1977) Growth and differentiation of normal and leukaemic human bone-marrow cells cultured in diffusion chambers. *Exp. Haematol.*, **5**, 199.

WINCHESTER, R. J., ROSS, G. D., JAROWSKY, C. I., WANG, C. Y., HALPER, J. & BROXMEYER, H. E. (1977) Expression of Ia-like antigen molecules on human granulocytes during early phases of differentiation. *Proc. Natl Acad. Sci. U.S.A.*, **74**, 4012.